# A premature stop codon within the *tvb* receptor gene results in decreased susceptibility to infection by avian leukosis virus subgroups B, D, and E

**DOI:** 10.18632/oncotarget.22512

**Published:** 2017-11-18

**Authors:** WeiGuo Chen, Yang Liu, Aijun Li, Xinjian Li, Hongxing Li, Zhenkai Dai, Yiming Yan, Xinheng Zhang, Dingming Shu, Huanmin Zhang, Wencheng Lin, Jingyun Ma, Qingmei Xie

**Affiliations:** ^1^ College of Animal Science, South China Agricultural University & Guangdong Provincial Key Lab of Agro-Animal Genomics and Molecular Breeding, Guangzhou 510642, P. R. China; ^2^ College of Science and Engineering, Jinan University, Guangzhou 510632, P. R. China; ^3^ Institute of Animal Science, Guangdong Academy of Agriculture Sciences, Guangzhou 510640, P. R. China; ^4^ USDA, Agriculture Research Service, Avian Disease and Oncology Laboratory, East Lansing, MI 48823, USA; ^5^ Key Laboratory of Animal Health Aquaculture and Environmental Control, Guangdong, Guangzhou 510642, P. R. China; ^6^ South China Collaborative Innovation Center for Poultry Disease Control and Product Safety, Guangzhou 510642, P. R. China

**Keywords:** avian leukosis virus, tvb receptor gene, premature stop codon, host resistance, chicken

## Abstract

Avian leukosis virus (ALV) is an oncogenic virus causing a variety of neoplasms in chickens. The group of avian leukosis virus in chickens contains six closely related subgroups, A to E and J. The prevalence of ALVs in hosts may have imposed strong selective pressure toward resistance to ALVs infection. The *tvb* gene encodes Tvb receptor and determines susceptibility or resistance to the subgroups B, D, and E ALV. In this study, we characterized a novel resistant allele of the *tvb* receptor gene, *tvb*^r3^, which carries a single-nucleotide substitution (c.298C>T) that constitutes a premature termination codon within the fourth exon and leads to the production of a truncated Tvb^R3^ receptor protein. As a result, we observed decreased susceptibility to infection by ALV-B, ALV-D and ALV-E both *in vitro* and *in vivo*, and decreased the binding affinity of the Tvb^R3^ receptor for the subgroups B, D, and E ALV envelope glycoproteins. Additionally, we found that the *tvb^r3^* allele was prevalent in Chinese broiler lines. This study demonstrated that premature termination codon in the *tvb* receptor gene can confer genetic resistance to subgroups B, D, and E ALV in the host, and indicates that *tvb^r3^* could potentially serve as a resistant target against ALV-B, ALV-D and ALV-E infection.

## INTRODUCTION

Avian leukosis viruses (ALVs) are members of avian retroviruses that induce diverse pathotypes of neoplastic diseases in chickens [1]. The spread of ALVs in poultry flocks worldwide has caused enormous economic losses [2]. Currently, there are no effective vaccines or drugs against ALVs. The eradication managements and biosecurity strategies are carried out to control ALVs infection [3]. However, these conventional methods cannot completely eliminate the spread of ALVs in chickens in China and Southeast Asia [4, 5]. A more effective antiviral strategy, therefore, may lie in breeding for resistance to infection by ALVs [6, 7].

The group of highly related avian leucosis viruses in chickens contains (A–E and J) six subgroups. ALVs infection is mediated by the interactions of viral envelope proteins with specific host receptors [8, 9]. The *tva*, *tvb,* and *tvc* loci in chickens encode the Tva, Tvb, and Tvc proteins, which are receptor for ALV-A, ALV-B/D/E, and ALV-C, respectively [10–14]. Two naturally occurring *tvb* susceptible alleles in chickens have been identified. The *tvb*^s1^ allele encodes the Tvb^S1^ receptor and confers susceptibility to ALV-B, ALV-D, and ALV-E, while the *tvb*^s3^ allele encodes Tvb^S3^ receptor and confers susceptibility to only ALV-B and ALV-D [15]. The chicken Na+/H+ exhanger type 1 (chNHE1), which is encoded by the *tvj* locus, was identified as the receptor for ALV-J [16]. In addition, chicken Annexin A2 (chANXA2) was also identified as a receptor for ALV-J [17]. The resistance to infection by a particular ALV subgroup can be caused by genetic alterations of specific receptor gene. Resistant alleles, *tva*^r^, *tvb*^r^, *tvc*^r^, and *tvj*^r^, have been identified in all four receptor loci. The genetic defects in the resistant alleles can result in premature stop codons, frame-shift mutation or intronic deletions in the receptor genes [14, 17–21], and substitutions of the critical amino acid residues in the receptor protein sequence [15, 22]. Additionally, the deletion of the tryptophan 38 in the first extracellular loop of NHE1 receptor accounts for the resistance to ALV-J in galliform species [23, 24]. Consequently, the resistant alleles not only confer host resistance to ALVs infection, but also provide valuable insight into antiviral strategies.

To further identify resistant alleles of ALV receptor genes, we screened the natural polymorphisms in specific receptor genes as a source of the host resistance in Chinese broiler lines [25]. In our previous study, we have identified two *tva* resistant alleles with decreased susceptibility to infection by ALV-A [21]. Here, we describe the decreased susceptibility to subgroups B, D, and E ALV in Chinese commercial broilers and respective molecular defect in *tvb* receptor gene.

## RESULTS

### Identification of the *tvb*^r3^ allele that introduces a premature stop codon

**Figure 1:**
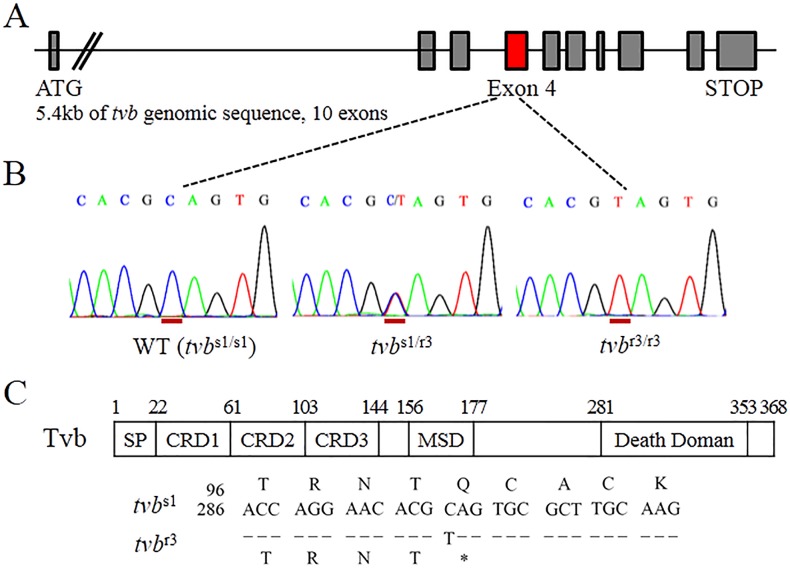
The *tvb*^r3^ allele in Chinese commercial broilers contains a premature stop codon **(A)** A schematic drawing of the structure of the *tvb* receptor gene. **(B)** Sequences traced from genomic DNA of homozygous wild-type (*tvb*^s1/s1^), heterozygous *tvb*^s1/r3^ and homozygous *tvb*^r3/r3^ birds covering c.294 to c.302 of *tvb*, showing the substitution of c.298C>T in the mutant, and the position of the substituted nucleotide is underlined in red. **(C)** Schematic of the Tvb protein is shown depicting the signal peptide (SP), cysteine-rich domains (CRDs), membrane spanning domain (MSD), and cytoplasmic death domain. The nucleotide and deduced amino acid sequences are shown surrounding the single nucleotide substitution that distinguishes *tvb*^r3^ from *tvb*^s1^ (numbered from the start methionine codon). The premature stop codon in *tvb*^r3^ is indicated by an asterisk.

In order to identify the natural mutations critical for resistance to subgroups B, D, and E ALVs infection, we amplified and sequenced the whole genomic region of the *tvb* receptor gene using 4 sets of specific primers (Supplementary Figure 1A), and dissected genetic mutations in the *tvb* receptor gene of a panel of 15 Chinese commercial broiler lines. During this process, we identified a single-nucleotide substitution within exon 4 of the *tvb* gene in Chinese chickens surveyed. In detail the sequence revealed the nucleotide change C>T located at 298 position of the *tvb* cDNA (c.298C>T) (Figure 1A and 1B). Since this c.298C>T substitution causes an in-frame stop codon in the open reading frame (ORF) of the *tvb* gene, implying a role in the resistance to subgroups B, D, and E ALVs infection [14, 18]. Thus, we designated this novel variant as *tvb*^r3^ allele. To confirm the c.298C>T substitution in *tvb*^r3^ transcript, we amplified the entire *tvb* coding sequence from the cDNA of *tvb*^s1/s1^ (wild-type) and *tvb*^r3/r3^ birds, and sequenced the RT-PCR products of the complete transcript (Supplementary Figure 1B). We then determined the nucleotide and deduced amino acid sequences of the *tvb* cDNA products and compared the nucleotide sequences of *tvb*^s1^ with the nucleotide sequences of *tvb*^r3^ allele. Only a single nucleotide difference was found, which changed the cytosine residue located 298 nucleotides downstream of the start methionine codon in *tvb*^s1^ to be a thymidine residue in *tvb*^r3^ (Figure 1C). Accordingly, these findings further indicated this single-nucleotide substitution changed codon 100 (CAG, glutamine) to a termination codon (UAG) in the Tvb^S1^ receptor protein.

### Mutant *tvb* mRNAs are targeted by the nonsense-mediated mRNA decay (NMD)

**Figure 2:**
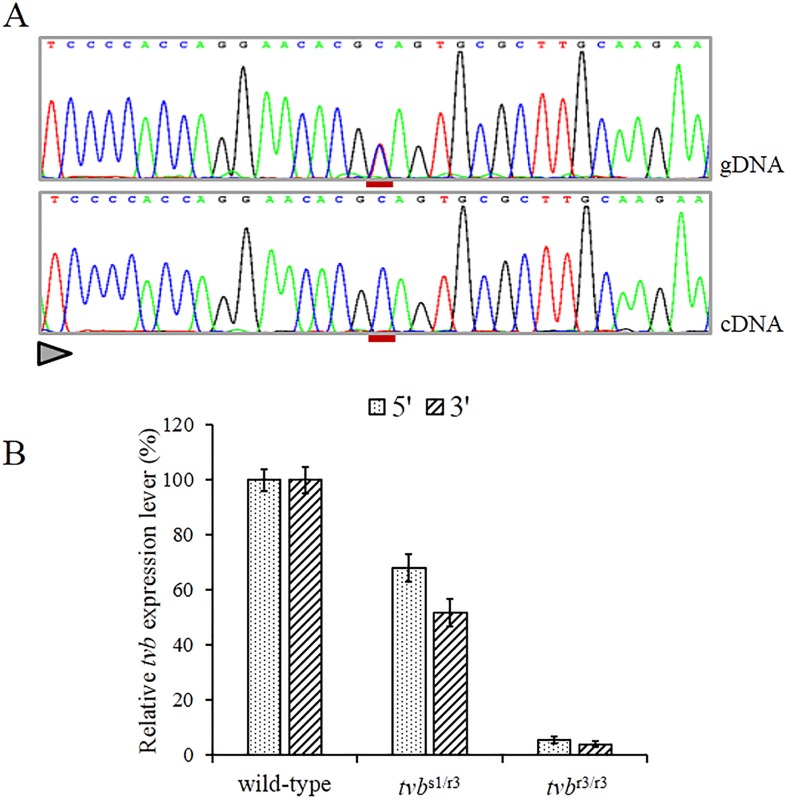
Nonsense-mediated RNA decay of c. 298C>T mutant *tvb* transcripts **(A)** Direct sequencing of *tvb* amplicons spanning the c.298C>T mutation obtained from genomic DNA and pulmonary cDNA of a heterozygous bird, showing the virtually exclusive detection of wild-type allele amongst transcripts (the position of the substituted nucleotide is underlined in red and the sequencing direction is represented by a triangle). **(B)** Comparing *tvb* mRNA levels in the blood of wild-type, *tvb*^s1/r3^ and *tvb*^r3/r3^ birds. Data are shown for two amplicons at the 5′ and 3′ ends of the *tvb* mRNA, respectively. Error bars correspond to standard errors over three replicates per sample.

Since the c.298C>T mutation causes a premature termination codon in the 4^st^ of the 10-exon *tvb* receptor gene. Thus, we assume that mutant *tvb* mRNAs may undergo NMD [26]. In order to verify this assumption, we firstly compared the *tvb* transcript levels between wild-type and mutant-type in blood of *tvb*^s1/r3^ birds by direct sequencing of RT-PCR products that encompassed the c.298C>T substitution. The results shown that mutant *tvb* mRNA was scarcely detectable (Figure 2A). Then, we compared the mRNA levels of *tvb* gene in blood of birds of *tvb*^s1/s1^, *tvb*^s1/r3^ and *tvb*^r3/r3^ genotypes using quantitative RT-PCR (qRT-PCR). In contrast to homozygous wild-type birds, highly significant reductions in *tvb* mRNA levels in *tvb*^s1/r3^ individuals were observed, 68%±5% and 52%±4% of control values for the 5′ and 3′ systems, respectively (Figure 2B). As for *tvb*^r3/r3^ birds, the *tvb* mRNA levels were less than 5% that of wild-types (Figure 2B). Altogether, the allelic imbalance test and qRT-PCR experiments both supported the notion that the mutant *tvb* transcripts were degraded by NMD.

### The *tvb*^r3^ allele generates a truncated Tvb receptor protein product

**Figure 3:**
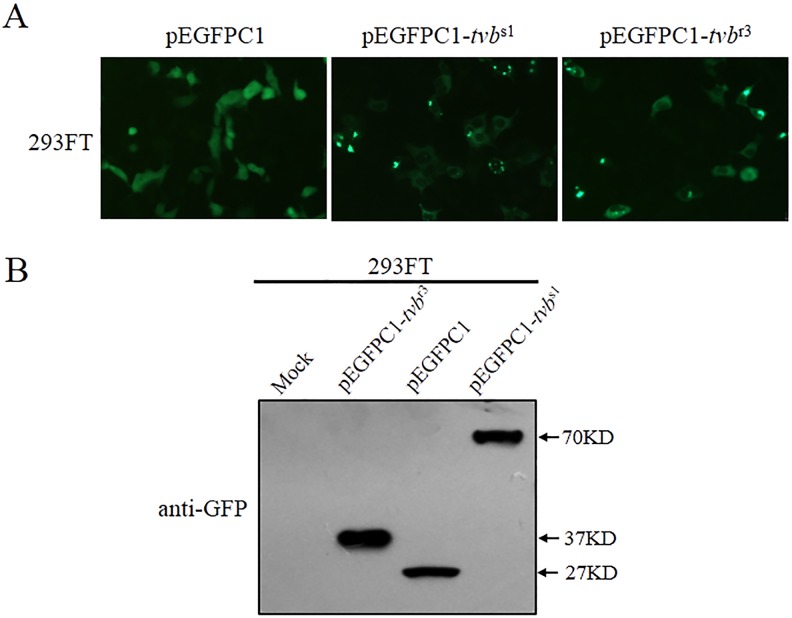
The *tvb*^r3^ generates a truncated Tvb^R3^ receptor protein product **(A)** 293FT cells were transfected with pEGFPC1, pEGFPC1-*tvb*^s1^, or pEGFPC1-*tvb*^r3^ plasmids DNA. Representative fields of view were captured at 24 h posttransfection with a fluorescence microscope (Scale bars: 100 μm). **(B)** 293FT cells were transfected with pEGFPC1 and pEGFPC1-*tvb* plasmids bearing the wt and mutant forms of Tvb, and cell lysates were subjected to SDS-PAGE and western blot analysis using the rabbit polyclonal antibody anti-GFP. The endogenous expression of human Tvb is shown in the cell lysates of mock-transfected cells. Sizes are indicated in kilodaltons.

Since the *tvb*^r3^ introduces a premature stop codon, it is predicted to generate a truncated Tvb receptor. To confirm this prediction, we estimated the effect of the *tvb*^r3^ allele on the expression of Tvb protein. To this aim, Tvb expression vectors with wild-type (pEGFPC1-*tvb*^s1^) and mutant *tvb* (pEGFPC1-*tvb*^r3^) were constructed and transfected into 293FT cells (Figure 3A). The expression of Tvb protein was analyzed by Western blotting analyses (Figure 3B). The GFP protein and Tvb/GFP fusion protein migrate as broad bands (27 to 70 KD). The band corresponding to Tvb^S1^/GFP fusion protein with a molecular weight of approximately 70 KD was observed (43 KD for Tvb^S1^ plus 27 KD for GFP). However, the band corresponding to Tvb^R3^/GFP fusion protein with a smaller molecular weight of ca.37 KD was detected (10 KD for Tvb^R3^ plus 27 KD for GFP). The endogenous Tvb expression was hardly visible, as the signal in mock-transfected 293FT cells (Figure 3B). Consequently, these results formally demonstrated that the *tvb*^r3^ allele generates a truncated Tvb^R3^ protein product at cysteine-rich domains 2 (CRD2) level (NP_989446.2:p.Thr99^*^).

### The *tvb*^r3^ allele reduces the susceptibility of Chinese chickens to infection by subgroups B, D, and E ALV *in vitro*

**Figure 4:**
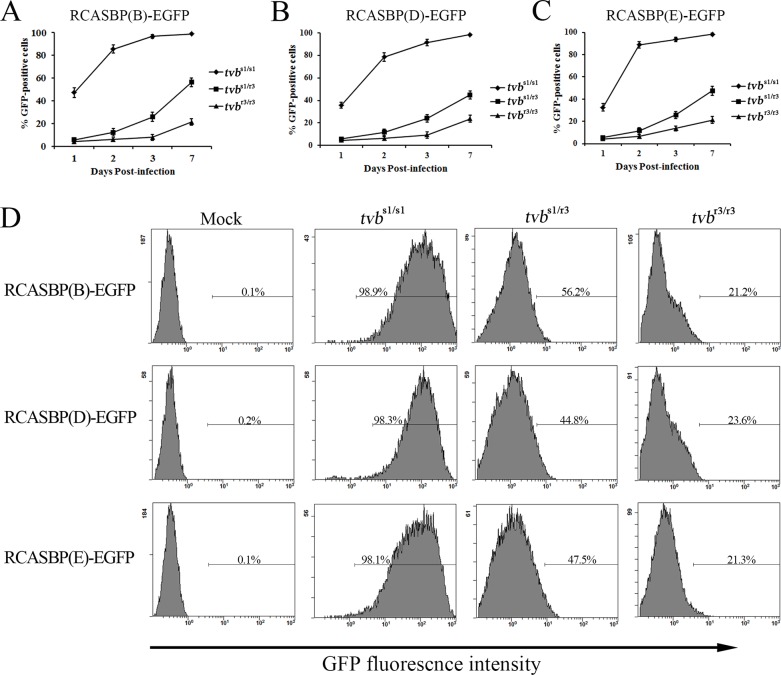
Time course of infection of *tvb*^s1/s1^, *tvb*^s1/r3^ and *tvb*^r3/r3^ CEFs with ALV reporter viruses CEFs of defined origin were infected at an MOI of 10 with replication-competent ALV recombinant reporter viruses, RCASBP(B)-EGFP **(A)**, RCASBP(D)-EGFP **(B)**, and RCASBP(E)-EGFP **(C)**. The percentage of GFP-positive cells was determined by FACS on indicated days postinfection, and the percentage of GFP-positive cells is indicated as a mean of three parallel dishes. **(D)** Representative FACS histograms of CEFs of defined origin infected with RCASBP(B)-EGFP, RCASBP(D)-EGFP and RCASBP(E)-EGFP at 7 days postinfection. The relative GFP fluorescence is plotted against the cell count and the percentage of GFP-positive cells is indicated.

To determine the effects of the *tvb*^r3^ allele on ALVs susceptibility, we first evaluated the infection and spread of the subgroups B, D, and E ALV reporter viruses in wild-type *tvb*^s1/s1^, *tvb*^s1/r3^ and *tvb*^r3/r3^ chicken embryo fibroblasts (CEFs). To this purpose, we constructed the RCASBP(B)-EGFP, RCASBP(D)-EGFP and RCASBP(E)-EGFP reporter vectors, transducing the enhanced green fluorescent protein (EGFP) reporter gene, based on the RCAS retrovirus vectors (Supplementary Figure 2A) [27]. The resulting replication-competent ALV recombinant viruses, RCASBP(B)-EGFP, RCASBP(D)-EGFP, and RCASBP(E)-EGFP were obtained as previously described (Supplementary Figure 2B) [21]. CEFs of different genotypes were infected with RCASBP(B)-EGFP, RCASBP(D)-EGFP, and RCASBP(E)-EGFP reporter viruses, and the time course of infection were followed as the percentage of GFP-positive cells quantified by fluorescence-activated cell sorting (FACS) on seven subsequent days. The *tvb*^s1/s1^ CEFs were used as a positive control since they are susceptible to subgroups B, D, and E ALV. As expected, RCASBP(B)-EGFP, RCASBP(D)-EGFP, and RCASBP(E)-EGFP infected the *tvb*^s1/s1^ CEFs efficiently, with almost one-half of the cells being infected by the day 1, and spread quickly, reaching virtually complete infection of cells on day 7 (Figure 4A, 4B, 4C). However, when the *tvb*^r3/r3^ CEFs were infected with RCASBP(B)-EGFP, RCASBP(D)-EGFP, or RCASBP(E)-EGFP reporter viruses, very different results were observed. RCASBP(B)-EGFP, RCASBP(D)-EGFP, and RCASBP(E)-EGFP infected the *tvb*^r3/r3^ CEFs inefficiently, with only 3.9%, 4.5% and 4.3% of the cells being infected on day 1, and the virus spread slowly, only approaching 21.2%, 23.6%, and 21.3% of infected cells on day 7 (Figure 4A, 4B, 4C). The GFP-negative and GFP-positive cells are obviously disparate, which were shown by the presence of two independent peaks in the FACS histogram (Figure 4D). We also determined the susceptibility of *tvb*^s1/r3^ CEFs to subgroups B, D, and E ALV reporter viruses, which exhibits an infection property between the *tvb*^r3/r3^ CEFs and its wild-type counterpart (Figure 4A, 4B, 4C).

**Figure 5:**
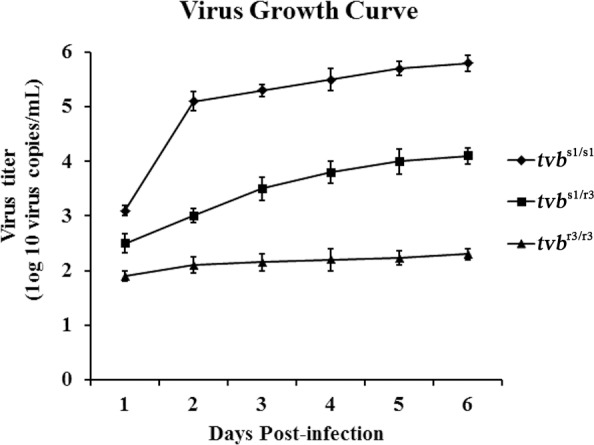
Growth curve of ALV-B wild virus in CEFs of defined origin The ALV-B strain SDAU09C2 growth curve was obtained by infection of *tvb*^s1/s1^, *tvb*^s1/r3^ and *tvb*^r3/r3^ CEFs at an MOI of 0.1. Cell culture supernatants associated viruses were harvested at 1, 2, 3, 4, 5, and 6 days postinfection, and titration experiments were performed using qPCR. Means ± standard deviations of data from three independent experiments performed in triplicates are shown.

In order to further verify these results, we next infected the CEFs with ALV-B wild virus and determined its growth kinetics in CEFs of defined origin by RT-PCR over a period of 6 days. The results showed that ALV-B strain SDAU09C2 has a significant growth advantage in *tvb*^s1/s1^ CEFs over in *tvb*^r3/r3^ CEFs, and a moderate growth advantage in *tvb*^s1/r3^ CEFs over in *tvb*^r3/r3^ CEFs (Figure 5). These findings were consistent with the infection by reporter viruses and suggested that the *tvb*^r3^ allele reduces the susceptibility of *tvb*^r3/r3^ CEFs to infection by ALV-B. Taken these results together, we clearly demonstrated that the *tvb*^r3^ allele resulted in inefficient infection and slow spread of subgroups B, D, and E ALV in *tvb*^r3/r3^ chickens *in vitro*.

### The *tvb*^r3^ allele reduces the susceptibility of Chinese chickens to infection by ALV-B *in vivo*

To further evaluate the effects of the *tvb*^r3^ allele on the infection and replication of the ALV-B *in vivo*, 1-day-old chicks were infected with ALV-B strain SDAU09C2 through the abdominal cavity. At one month postinfection, the infection status of ALV-B in each chick was determined. As shown in Table 1, the *tvb*^r3/r3^ chicks showed a significantly lower infection proportion than that of *tvb*^s1/s1^ chicks, and a lower infection proportion than that of *tvb*^s1/r3^ chicks. In contrast to 100% of infected *tvb*^s1/s1^ chicks and 50% of infected *tvb*^s1/r3^ chicks, only 26.3% of the *tvb*^r3/r3^ chicks being infected by ALV-B strain SDAU09C2. These results demonstrated that the *in vivo* infection and replication coincides with spread of the virus *in vitro*.

**Table 1:** Incidence of the infection by subgroup B ALV

	Genotype	No. of positive samples/total no. of samples	Positive infection (%)
Commercial chicks	*tvb*^s1/s1^	10/10	100
	*tvb*^s1/r3^	8/16	50
	*tvb*^r3/r3^	5/19	26.3
SPF chicks	*tvb*^s1/s1^	6/6	100

### The *tvb*^r3^ allele decreases the binding affinity for the ALV envelope glycoproteins

**Figure 6:**
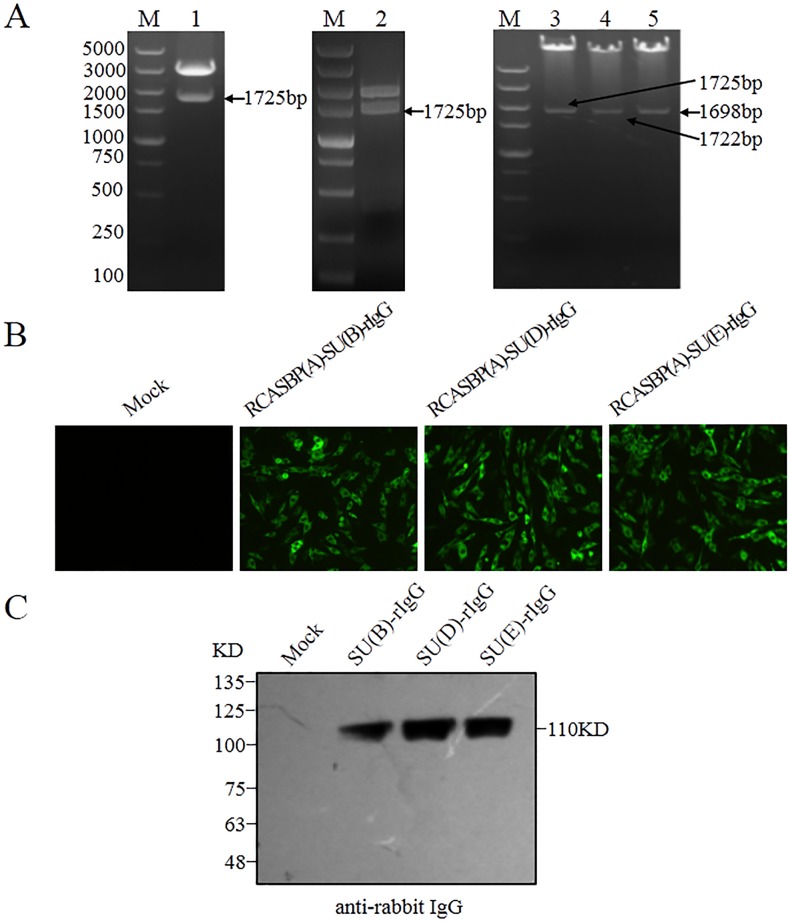
Construction and expression of SU(B)-rIgG, SU(D)-rIgG and SU(E)-rIgG immunoadhesin **(A)** Restriction enzyme digestion analysis of recombinant plasmids pUC18-SU(B)-rIgG (Lane 1), Cla12Nco-SU(B)-rIgG (Lane 2), and RCASBP(A)-SU(B)-rIgG (Lane 3), RCASBP(A)-SU(D)-rIgG (Lane 4), RCASBP(A)-SU(E)-rIgG (Lane 5). M: DNA Marker 5000, the sizes of diagnostic fragment which fused ALV SU gene and the Fc region of rabbit IgG are indicated on the left, and SU(B), SU(D) and SU(E) represent the ALV-B, ALV-D, and ALV-E SU gene, respectively. **(B)** DF-1 cells were transfected with RCASBP(A)-SU(B)-rIgG, RCASBP(A)-SU(D)-rIgG and RCASBP(A)-SU(E)-rIgG plasmids DNA or with no DNA (mock). The expression of SU(B)-rIgG, SU(D)-rIgG and SU(E)-rIgG immunoadhesin proteins were detected by direct immunofluorescence assay, and Alex Fluor 488-goat anti-rabbit IgG was used as the antibody. Representative fields of view were captured at 48 h posttransfection with an inverted fluorescence microscope (Scale bars: 100 μm). **(D)** Western immunoblot analysis of the soluble forms of the SU glycoproteins SU(B)-rIgG, SU(D)-rIgG, and SU(E)-rIgG immunoprecipitated with anti-rabbit IgG-agarose beads. The precipitated proteins were denatured and separated by SDS-PAGE, and transferred to nitrocellulose. The filters were probed with horseradish peroxidase-conjugated goat anti-rIgG, and the bound protein-antibody complexes were visualized by chemiluminescence. Molecular sizes (in kilodaltons) are given.

**Figure 7:**
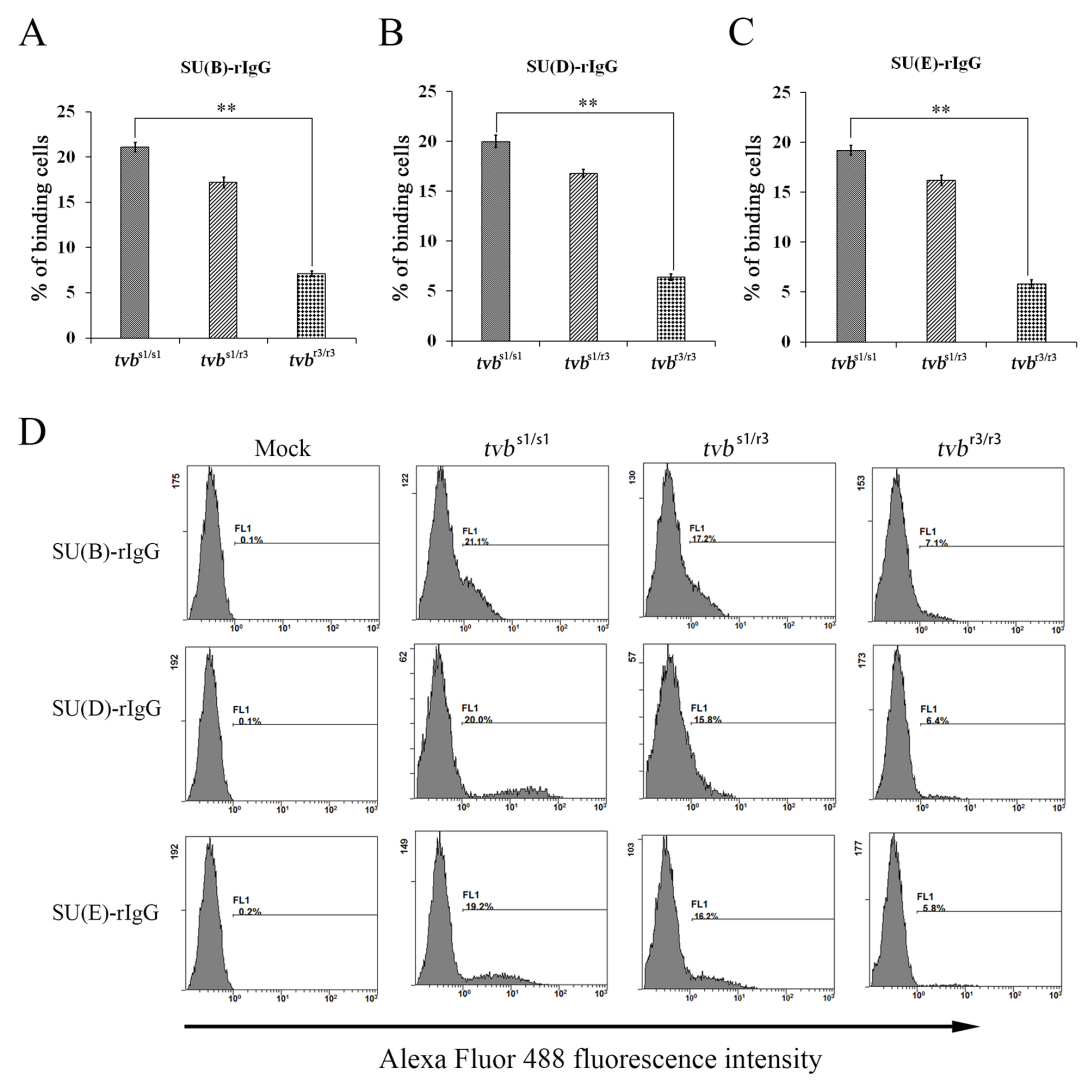
Binding affinities of ALV envelope glycoproteins for Tvb receptors expressed on the surfaces of CEFs of defined origin The *tvb*^s1/s1^, *tvb*^s1/r3^ and *tvb*^r3/r3^ CEFs were incubated with the same amount of soluble forms of the SU-rIgG, SU(B)-rIgG **(A)**, SUD-rIgG **(B)** or SUE-rIgG **(C)**. The viral envelope glycoprotein-receptor complexes were bound to goat-anti-rabbit IgG conjugated with Alexa Fluor 488, and the amount of Alexa Fluor 488 bound to the cells was quantitated by FACS. Binding affinities of the envelope-receptor is given as the percentage of fluorescence-positive cells. The values shown are means ± standard deviation from three independent experiments. To determine the statistical significance of differences between binding of wt and the mutant Tvb receptors for all three soluble SU glycoproteins data sets, the Student's *t* tests were performed (^**^, *P*< 0.01). **(D)** Representative examples of FACS histograms showing the percentages of Alexa Fluor 488-positive cells after incubation of *tvb*^s1/s1^, *tvb*^s1/r3^ and *tvb*^r3/r3^ CEFs with the same amount of each soluble forms of the SU-rIgG and negative controls incubated without immunoadhesin.

Mutations in *tvb* gene may either abrogate or lower virus envelope-receptor binding affinity. To test this notion, chimeric immunoadhesins SU(B)-rIgG, SU(D)-rIgG and SU(E)-rIgG, which composed of the subgroups B, D and E-specific surface (SU) glycoprotein fused to the constant fragment of rabbit IgG, were constructed (Figure 6A), and transfected into DF-1 cells. The expression of fusion proteins SU(B)-rIgG, SU(D)-rIgG and SU(E)-rIgG was detected by direct immunofluorescence assay using Alex Fluor-conjugated goat anti-rabbit IgG antibody (Figure 6B). The integrity of the immunoadhesin proteins SU(B)-rIgG, SU(D)-rIgG and SU(E)-rIgG was verified by Western immunoblot analysis (Figure 6C). The binding of the SU(B)-rIgG, SU(D)-rIgG or SU(E)-rIgG for Tvb receptors expressed on the surfaces of *tvb*^s1/s1^, *tvb*^s1/r3^ and *tvb*^r3/r3^ CEFs were detected by fluorescein-conjugated goat anti-rabbit antibody and determined by flow cytometry as described previously [14, 23]. As excepted, the SU(B)-rIgG, SU(D)-rIgG or SU(E)-rIgG fusion proteins bound the Tvb^S1^ receptor expressed on the surfaces of *tvb*^s1/s1^ CEFs efficiently, with 21.1%, 20% and 19.2% *tvb*^s1/s1^ CEFs being bound at 2 h after incubation (Figure 7A, 7B, 7C). However, binding of all three soluble SU proteins to the Tvb^R3^ receptor expressed on the surfaces of *tvb*^r3/r3^ CEFs with significantly lower affinities than those of Tvb^S1^ receptor (*p*<0.01), at 3- to 4-fold lower affinity, with only 7.1%, 6.4% and 5.8% *tvb*^r3/r3^ CEFs being bound (Figure 7A, 7B, 7C). The fluorescein-negative and fluorescein-positive cells are obviously disparate, which were shown by the presence of two independent peaks in the FACS histogram (Figure 7D). Collectively, these findings provided key evidence that the reduction in the binding affinity of Tvb^R3^ is relevant to the decreased infection efficiency of ALV-B, ALV-D, and ALV-E in *tvb*^r3/r3^ CEFs.

### The *tvb*^r3^ allele are widely distributed in Chinese commercial broilers

Finally, we investigated the distribution of *tvb*^r^ [18], *tvb*^r2^ [22] and *tvb*^r3^ alleles in Chinese commercial broiler lines. To this end, the sequence of the *tvb* genomic region, including these 3 resistant alleles, was amplified and sequenced. Sequencing revealed that the genotypes and their frequency among these 3 resistant alleles were obviously different in Chinese broiler lines (Table 2). Compared to *tvb*^r^ and *tvb*^r2^ alleles, *tvb*^r3^ was prominently distributed in Chinese commercial broilers, suggesting that the *tvb*^r3^ allele can used to be a biomarker to improve the resistance of Chinese chickens to infection by ALV-B, ALV-D, and ALV-E through genetic selection.

**Table 2:** Genotypic frequency of resistant alleles in the *tvb* receptor gene in Chinese commercial broiler lines

Line^a^	No.	*tvb*^r b^			*tvb*^r2 c^			*tvb*^r3^		
		*tvb*^s1/s1^	*tvb*^s1/r1^	*tvb*^r1/r1^	*tvb*^s1/s1^	*tvb*^s1/r2^	*tvb*^r2/r2^	*tvb*^s1/s1^	*tvb*^s1/r3^	*tvb*^r3/r3^
CB01	25	1	0	0	0.92	0	0.08	0.36	0.24	0.40
CB02	30	1	0	0	1	0	0	0	0.10	0.90
CB03	26	1	0	0	1	0	0	0.15	0.46	0.39
CB04	31	1	0	0	1	0	0	0.13	0.13	0.74
CB05	26	1	0	0	1	0	0	0	0.12	0.88
CB06	29	1	0	0	1	0	0	0	0	1
CB07	31	0.77	0.2	0.03	1	0	0	0.48	0.32	0.19
CB08	30	1	0	0	1	0	0	0.33	0.27	0.40
CB09	31	1	0	0	1	0	0	0.16	0.39	0.45
CB10	29	1	0	0	1	0	0	0	0.10	0.90
CB11	30	1	0	0	0.77	0.16	0.07	0.53	0.27	0.20
CB12	30	1	0	0	1	0	0	0.10	0.37	0.53
CB13	30	1	0	0	1	0	0	0.20	0.47	0.33
CB14	36	0.61	0.33	0.06	1	0	0	0.56	0.11	0.33
CB15	30	0.60	0.23	0.17	1	0	0	0.60	0.40	0.00

^a^ CB01, CB02, CB03, CB04, CB05, CB06, CB07, CB08, CB09, CB10, CB11, CB12, CB13, CB14 and CB15 represent the commercial broiler line 01, 02, 03, 04, 05, 06, 07, 08, 09, 10, 11, 12, 13, 14 and 15, respectively.

^b^ The *tvb*^r^ allele contains a premature stop codon at codon 58 in Tvb^S1^ receptor, which explains the resistance to infection by ALV-B, ALV-D and ALV-E [23].

^c^ The *tvb*^r2^ allele encodes a mutant Tvb^S1^ receptor with the C125S substitution in CRD3, reduces the susceptibility to infection by ALV-B and ALV-D and virtually eliminates susceptibility to ALV-E infection [27].

## DISCUSSION

We previously identified intronic deletions within the *tva* receptor gene resulted in reduced susceptibility to subgroup A ALV [21], which implies the presence of similar genetic mutations within other ALV receptor genes. Indeed, we herein identified that the c.298C>T substitution in the *tvb* gene reduces the susceptibility of Chinese commercial broilers to subgroups B, D, and E ALV infection *in vitro* and *in vivo*. This is the first example to report a genetic mutation in the *tvb* gene that accounts for the quantitative effect on susceptibility to ALV-B, ALV-D, and ALV-E in Chinese chickens.

Premature termination codons (PTCs), which could result from nonsense mutations or frame-shifts, lead to a reduction of mRNA level. This phenomenon is known as nonsense-mediated mRNA decay (NMD), which generally requires that PTCs at least located 55 bases upstream of the last exon-exon junction [28]. Thus, the PTC in the *tvb*^r3^ transcript located 73 bases upstream of the exon 4-exon 5 junction may be a proper target. We assume that aberrant *tvb* mRNAs results from the c.298C>T substitution may be targeted by NMD. The quantification of gene expression results showed a minimal *tvb* expression compared to the normal expression (Figure 2). This aspect is consistent with the role of NMD in degrading mRNAs that contain a premature termination codon. Thus, in our case, NMD pathway acts in the control of *tvb* receptor gene expression by strongly reducing the aberrant mRNAs.

Some specific regions and residues in the Tvb receptor have been identified to be crucial to efficiently mediate ALV-B, ALV-D and ALV-E infection. The Tvb_32-46_ domain in the CRD1 was identified as a minimal soluble Tvb receptor, and residues Leu-36, Gln-37, Leu-41, and Tyr-42 in this region have been reported to be the interaction determinants for ALV-B and ALV-D [29]. By contrast, CRD1, CRD2, and CRD3 of Tvb receptor are required for binding and entry of ALV-E [15, 22, 30]. The structural integrity of the Tvb protein, including residues Tyr-67, Asn-72, and Asp-73 in CRD2, are crucial for proper receptor function [30]. The *tvb*^s3^ allele encodes Tvb^S3^ receptor, which contains the substitution of Cys62Ser, likely alters the folding and final structure of the CRD2, abrogating the binding and entry of ALV-E [15]. Published study has also demonstrated the Tvb^R2^ protein with the substitution of Cys125Ser in CRD3, presumably alters the structure of at least CRD3, and may further lead to alterations in other CRDs structure of the Tvb^R2^ protein, such as CRD1 and CRD2, significantly reducing the binding and entry of ALV-B and ALV-D, and virtually eliminating the protein functions as a receptor for ALV-E [22]. Since the c.298C>T substitution in *tvb* receptor gene, namely *tvb*^r3^ allele, generates a truncated Tvb^R3^ protein, which consists of only the 99 N-terminal amino acid residues (Figure 1C). As discussed above, it seems obvious to propose that this c.298C>T substitution may exert the same effects and also cause alterations in structure of the Tvb^S1^ receptor protein. In this scenario, the c.298C>T substitution likely alters the final structure of CRD2, and may interfere with and/or alter the structure of CRD1. Alternatively, the c.298C>T substitution may cause an alteration in the structures of both CRD1 and CRD2. Therefore, if the c.298C>T mutation alters both CRD2 and CRD1, thereby changing the structure of the Tvb protein in a way that reduces but not eliminates ALV-B, ALV-D and ALV-E interaction and subsequent virus entry, which could be plausible explanation for the phenotype of Tvb^R3^ protein.

From the point of view of coevolution between ALVs and host, it is attractive to speculate that the *tvb*^r3^ may evolve from the *tvb*^s1^ allele during the selective breeding of Chinese commercial broiler lines to confer resistance to infection by subgroups B, D, and E ALV [31]. As a consequence, the *tvb*^r3^ allele is widespread in the commercial chicken populations. In fact, the *tvb*^r3^ allele was prevalent in Chinese broiler lines (Table 1), which is consistent with the speculation. Consequently, the *tvb*^r3^ allele can used to be a biomarker for breeding chickens genetically resistance to subgroups B, D, and E ALV.

In summary, the present study identified a resistant allele *tvb*^r3^ in Chinese commercial broilers. The *tvb*^r3^ allele contains a novel c.298C>T substitution, which introduces a premature stop codon and results in the generation of the truncated Tvb^R3^ protein, significantly reducing the binding to the ALV-B, ALV-D and ALV-E glycoproteins and virus infection efficiency. These findings provide potential target for the development of host resistance to ALV-B, ALV-D and ALV-E infection.

## MATERIALS AND METHODS

### Experimental animals

The Chinese commercial broiler line 01 to 15 (CB01 to CB15) have been maintained at Guangdong Wen's Food Group Co., Ltd [25]. Fertilized eggs were incubated at 37.8°C and 58% relative humidity in a forced-air incubator with a tilting motion through a 90° angle every 2 h. All animal experiments were performed following protocols approved by the Institutional Animal Care and Use Committee at the South China Agricultural University, China (approval ID: 201004152).

### Cell culture and virus preparation

The procedure for the preparation of CEFs from ten-day-old embryos from *tvb*^s1/s1^, *tvb*^s1/r3^ and *tvb*^r3/r3^ fertilized eggs has been described previously [32]. CEFs, DF-1 and 293FT cells were grown in Dulbecco's modified Eagle's medium (DMEM; Invitrogen Gibco, Carlsbad, CA, USA) supplemented with 10% fetal bovine serum (FBS; Invitrogen Gibco) and penicillin/streptomycin (100 mg/ml each) at 37°C under 5% CO2. The ALV-B strain SDAU09C2 (kindly provided by Professor Zhizhong Cui of Shandong Agricultural University) was propagated in DF-1 cells. The value of the ratio of the sample to the positive control (S/P) for ALV p27 antigen of ALV-B strain SDAU09C2 was determined by an avian leukosis virus antigen test kit (IDEXX, Inc., Westbrook, MA).

### DNA extraction, RNA isolation and cDNA synthesis

Genomic DNA was extracted from blood samples collected from lines CB01 to CB15 chickens, and chicks infected by ALV-B strain SDAU09C2 using phenol-chloroform protocol. The proviral DNA of strain SDAU09C2 was extracted as described previously [33]. The RCASBP(A)-EGFP, RCASBP(B), RCASBP(D) and RCASBP(E) plasmids DNA were extracted using DNA Extraction Kits for Plasmids (Bio-Rad Laboratories, Inc). Total RNA was extracted from blood samples collected from the rabbit, *tvb*^s1/s1^, *tvb*^s1/r3^, *tvb*^r3/r3^ birds, as well as chicks and CEFs infected by ALV-B strain SDAU09C2 using Trizol reagent (Invitrogen). cDNA was obtained by reverse transcription of 1 μg of DNA-free RNA using ReverTra Ace® qPCR RT Kit (Toyobo, Tokyo, Japan). The prepared DNA and synthesized cDNA were stored at −20°C for further processing.

### Primer design

PCR primers that were specific for the whole genomic region of *tvb* gene sequence clone, the full-length *tvb* coding sequence clone and a portion of *tvb* cDNA clone encompassing the c.298C>T mutation, as well as RT-qPCR primers that were specific for 5′ and 3′ end of the *tvb* gene, and chicken glyceraldehyde-3-phosphate dehydrogenase (chGAPDH) were designed using Premier Primer 5.0 software (Premier Biosoft, Palo Alto, CA, USA) (Supplementary Table 1). PCR primers that were specific for the gp85 (SU) coding region of subgroups B, D and E ALV sequence clone and the IgG heavy chain of rabbit clone were also designed using Premier Primer 5.0 software (Supplementary Table 2). All the above primers were synthesized by Sangon Biotech Co., Ltd (Guangzhou, China).

### Amplification and analysis of *tvb* alleles from commercial broiler lines

The sequence of the whole *tvb* gene genomic region was amplified from genomic DNA of a panel of 15 commercial broiler lines using four specific primer pairs with KOD FX (Toyobo, Tokyo, Japan). The PCR products were directly sequenced by Sangon Biotech Co., Ltd. Four *tvb* fragments sequence traces from Chinese broiler lines were aligned and compared using Lasergene version 7.1 (DNAStar, Inc., Madison, WI). The sequence of the *tvb* gene genomic region that contains *tvb^r^*, *tvb^r2^* and *tvb^r3^* alleles was amplified using *tvb*-3 primer pair, and sequenced directly. In total, *tvb^r^*, *tvb^r2^* and *tvb^r3^* alleles of 444 birds from these 15 commercial broiler lines were genotyped.

### Transcript analysis of *tvb* alleles by RT-PCR

The whole *tvb* coding sequence was amplified from the cDNA of *tvb*^s1/s1^ or *tvb*^r3/r3^ birds using specific primers with KOD-Plus-Neo (Toyobo, Tokyo, Japan). In order to identify the transcripts of the *tvb*^s1/s1^ or *tvb*^r3/r3^ genotypes, the RT-PCR products were visualized by electrophoresis on 2% agarose gels, and then ligated into the pMD19-T vector (TaKaRa, Dalian, China) for sequencing.

### Allelic imbalance test and qRT-PCR test of NMD

A portion of *tvb* cDNA encompassing the c.298C>T mutation, was amplified from cDNA of *tvb*^s1/r3^ birds. The RT-PCR products were ligated into the pMD19-T vector (TaKaRa) and then sequenced. To detect the mRNA expression level associated with 5′ and 3′ end of the *tvb* gene in the *tvb*^s1/s1^, *tvb*^s1/r3^ and *tvb*^r3/r3^ birds. qRT-PCR were performed using an ABI7500 instrument (Applied Biosystems, Foster City, CA, USA) with FastStart SYBR Green Master (Rox) (Roche, Switzerland). The chicken *GAPDH* was used as an internal control. All samples were analyzed in triplicate. The analysis was carried out using the 2^−ΔΔ*CT*^ method as previously described [34].

### Construction of the *tvb* expression vectors and Western blot analysis

The entire *tvb* coding sequence was amplified from the cDNA of *tvb*^s1/s1^ or *tvb*^r3/r3^ birds, and cloned into the pEGFPC1 vector (Clontech, USA) with the proper orientation by *Xho*I and *Hind*Ш (New England BioLabs) digestion. The resulting expression constructs with wt (pEGFPC1-*tvb*^s1^) and mutated *tvb* (pEGFPC1-*tvb*^r3^), as well as pEGFPC1 and empty vectors were transfected into 293FT cells by Lipofectamine 3000 reagent (Invitrogen). At 48 h posttransfection, 293FT cells were subjected to Western blot analysis as previously described [35]. Briefly, the rabbit polyclonal antibody anti-GFP (1:1000; Cell Signaling Technology) was used as the primary antibody, and horseradish peroxidase (HRP)-conjugated goat anti-rabbit IgG (1:8,000; Proteintech Group, Inc., USA) was used as the secondary antibody.

### Construction of subgroups B, D, E ALV reporter vectors and virus propagation

The replication-competent ALV recombinant virus RCASBP(A)-EGFP, transducing the enhanced green fluorescent protein (EGFP) reporter gene, has been described previously [21]. The *EGFP* gene was isolated from RCASBP(A)-EGFP as *Cla*I (New England BioLabs) fragment and cloned into the *Cla*I site of the RCASBP(B), RCASBP(D), and RCASBP(E) retroviral vectors [27], which were kindly obtained from Stephen H. Hughes (HIV Drug Resistance Program, National Cancer Institute, USA). The resulting expression constructs RCASBP(B)-EGFP and RCASBP(D)-EGFP were transfected into DF-1 cells using JetPRIME Regent (Invitrogen). The RCASBP(E)-EGFP was transfected into CEFs prepared from 10-day-old embryos of specific-pathogen-free (SPF) chickens using Lipofectamine 3000 reagent (Invitrogen). Infection and spread of reporter virus were observed as an increasing proportion of GFP-positive cells, and virus stocks were harvested on day 7 posttransfection. Virus stocks were generated from cell supernatants cleared of cellular debris by centrifugation at 2,000×g for 10 min at 4°C and stored in aliquots at −80°C. The titers were quantitated by terminal dilution and infection of fresh DF-1 cells, reached 10^6^ infection units (IU) per ml.

### Virus spread assayed by flow cytometry

The *tvb*^s1/s1^, *tvb*^s1/r3^ and *tvb*^r3/r3^ CEFs were seeded in triplicate wells at a density of 5×10^4^ per well in a 24-well plate, and infected with 5×10^5^ IU of either RCASBP(B)-EGFP, RCASBP(D)-EGFP or RCASBP(E)-EGFP viruses 24 h after seeding. After incubation for 1 h, the cells were overlaid with DMEM supplemented with 1% FBS and incubated at 37°C in a 5% CO2 atmosphere for 7 days. The percentage of GFP-positive cells was quantitated by FACS using a Cytomics FC 500 analyzer (Beckman Coulter, Kurt, U.S.A) on days 1, 2, 3 and 7 postinfection. The cells of three wells were trypsinized and washed in phosphate-buffered saline (PBS) before the analysis.

### Virus growth kinetics of subgroup B ALV

The *tvb*^s1/s1^, *tvb*^s1/r3^ and *tvb*^r3/r3^ CEFs were seeded in triplicate wells at a density of 1×10^6^ per well in a 6-well plate. 24 h after seeding, CEFs were infected with ALV-B strain SDAU09C2 (S/P: 2.0) at a multiplicity of infection (MOI) of 0.1. After 1h the medium was replaced by fresh medium, and the infected cell cultures were harvested at days 1, 2, 3, 4, 5, and 6 postinfection, which were subjected to RNA extraction and cDNA synthesis. The titer of infectious progeny was determined as the virus copies per milliliter by real-time PCR using ALV-B specific primers pair as previously described [36]. The *GAPDH* gene was used as an internal control. The mean values and standard deviations were calculated from three independent experiments.

### Experimental infections

45 1-day-old chicks randomly collected from commercial broiler lines were randomly divided into three groups, with 15 chicks and 2 SPF chicks each group. Chicks were maintained in three negatively-pressured biosecurity isolators under quarantine conditions and provided with water and feed *ad libitum.* Chicks were inoculated with 0.3 ml of ALV-B strain SDAU09C2 (2.2 S/P) through the abdominal cavity, and inoculated once again at 5 days old. Genomic DNA from each chick at 7 days old was used to genotype the *tvb*^r3^ allele by direct sequencing. At one month postinfection, the status of infection in each chick by ALV-B strain SDAU09C2 was determined using the method as previously described [37].

### Construction of SU(B)-rIgG, SU(D)-rIgG and SU(E)-rIgG and production of immunoadhesin

The gp85 (SU) coding region of ALV-B, ALV-D and ALV-E were amplified by PCR from the isolate SDAU09C2 provirus DNA, RCASBP(D) and RCASBP(E) plasmids DNA, respectively. The IgG heavy chain of rabbit (rIgGFc) was obtained by RT-PCR from the RNA of rabbit blood cells. The SU(B) fragment and the rIgGFc were ligated with *Sac*I/*Kpn*I (New England BioLabs) and *Kpn*I/*BamH*I (New England BioLabs) restriction enzyme sites, and the resulting fusion fragment was subcloned into the pUC18 vector (Promega, Madison, USA), to generate the pUC18-SU(B)-rIgG vector containing a ca. 1.8kb fragment which fused ALV-B SU and rIgGFc genes. After that, we cloned SU(B)-rIgG into the Cla12Nco adapter plasmid to generate the Cla12Nco-SU(B)-rIgG vector by *Sac*I and *BamH*I sites. The recombinant Cla12Nco-SU(D)-rIgG and Cla12Nco-SU(E)-rIgG vectors were constructed by replacing the SU(D)-rIgG and SU(E)-rIgG sequence in Cla12Nco-SU(B)-rIgG using the *Sac*I and *BamH*I restriction sites. The SU(B)-rIgG, SU(D)-rIgG and SU(E)-rIgG sequence were then cloned into RCASBP(A) vector as the *Cla*I fragment. The final recombinant vectors RCASBP(A)-SU(B)-rIgG, RCASBP(A)-SU(D)-rIgG and RCASBP(A)-SU(E)-rIgG were transfected into DF-1 cells. The cell supernatants that expressed immunoadhesin proteins SU(B)-rIgG, SU(D)-rIgG or SU(E)-rIgG were cleared by centrifugation at 2,000 × g for 10 min at 4°C, and then stored at −80°C in aliquots. The levels of all three soluble SU proteins were quantitated by enzyme-linked immunosorbent assay (ELISA) for the rabbit IgG tag as previously described [38].

### Direct immunofluorescence assay and Western immunoblot analysis

Direct immunofluorescence assays were carried out on the DF1 cells at 48 h posttransfection, and Alex Fluor-goat anti-rabbit IgG (1:8,000; Invitrogen) were used as the antibody. At 48 h posttransfection, the immunoadhesin proteins SU(B)-rIgG, SU(D)-rIgG and SU(E)-rIgG were analyzed by Western immunoblotting as previously described [14, 39]. The horseradish peroxidase-conjugated goat anti-rIgG (1:10,000; Proteintech Group, Inc., USA) was used as the secondary antibody.

### Binding affinity was analyzed by flow cytometry

The *tvb*^s1/s1^, *tvb*^s1/r3^ and *tvb*^r3/r3^ CEFs were seeded in triplicate wells at a density of 2×10^5^ per well in a 6-well plate. After reaching about 90% confluence, CEFs of defined origin were harvested by 0.25% trypsin solution, and washed in PBS supplemented with 2% calf serum (PBS-CS), centrifuged for 5 min at 500×g, and resuspended in 200 μL of PBS-CS. The cells in PBS-CS were incubated with supernatant containing SU(B)-rIgG, SU(D)-rIgG or SU(E)-rIgG fusion protein with the same concentration (10 ng of rIgG Fc fragment per ml) on ice for 1 h. After three washes with PBS-CS, the goat anti-rabbit IgG linked to Alexa Fluor 488 (Invitrogen) was diluted 1:100 in PBS supplemented with 4% calf serum, and washed cells were incubated in 500 μL of diluted antibody on ice for 30 min. After incubation and three washes in PBS-CS, the complexes of cells with immunoadhesins were resuspended in 200 μL PBS-CS, and the percentage of fluorescence-positive cells was quantitated by FACS using a Cytomics FC 500 analyzer (Beckman Coulter, Kurt, U.S.A).

### Statistical analysis

All experiments were performed with at least three independent replicates. Differences in data were evaluated by the Student's t test. A *p* value of < 0.05 was considered a significant difference between the groups.

## SUPPLEMENTARY MATERIALS FIGURES AND TABLES


