# Supplementary material for: A premature stop codon within the tvb receptor gene results in decreased susceptibility to infection by avian leukosis virus subgroups B, D, and E
Source: Oncotarget. 2017 Nov 18;8(62):105942–56. doi: 10.18632/oncotarget.22512 (PMC5739692; doi:10.18632/oncotarget.22512)
Supplement: Supplementary file 1 [file oncotarget-08-105942-s001.pdf]

A premature stop codon within the *tvb* receptor gene results in decreased susceptibility to infection by avian leukosis virus subgroups B, D, and E

SUPPLEMENTARY MATERIALS

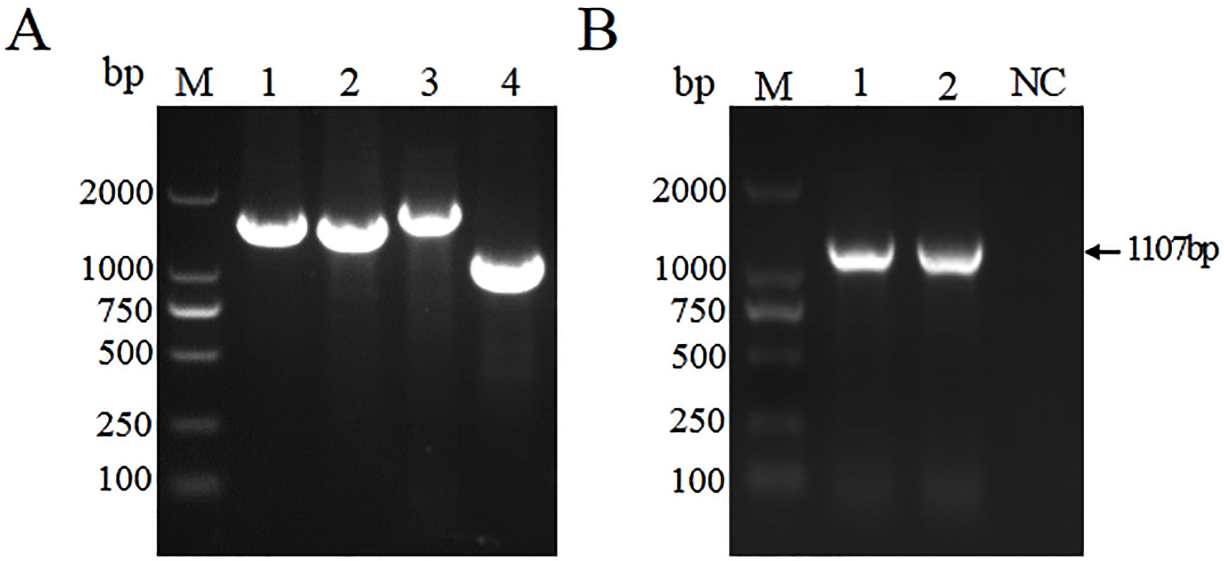

Supplementary Figure 1: Sequence amplification of *tvb* receptor gene. (A) PCR amplification of the whole genomic region of the *tvb* gene. M: DNA Marker 2000; Lane 1, 2, 3, 4: PCR products amplified by *tvb*-1, *tvb*-2, *tvb*-3 and *tvb*-4 primer pairs, with a size of 1495bp, 1486bp, 1625bp and 1055bp, respectively. **(B)** RT-PCR amplification of the entire coding sequence of the *tvb* gene. M: DNA Marker 2000; Lane 1, 2: RT-PCR products from blood samples of the *tvb*$^{s1/s1}$ and *tvb*$^{r3/r3}$ birds, the sizes of diagnostic PCR products are indicated on the left. NC: negative control. The gels have been run under the same experimental conditions, and the cropped gels are used.

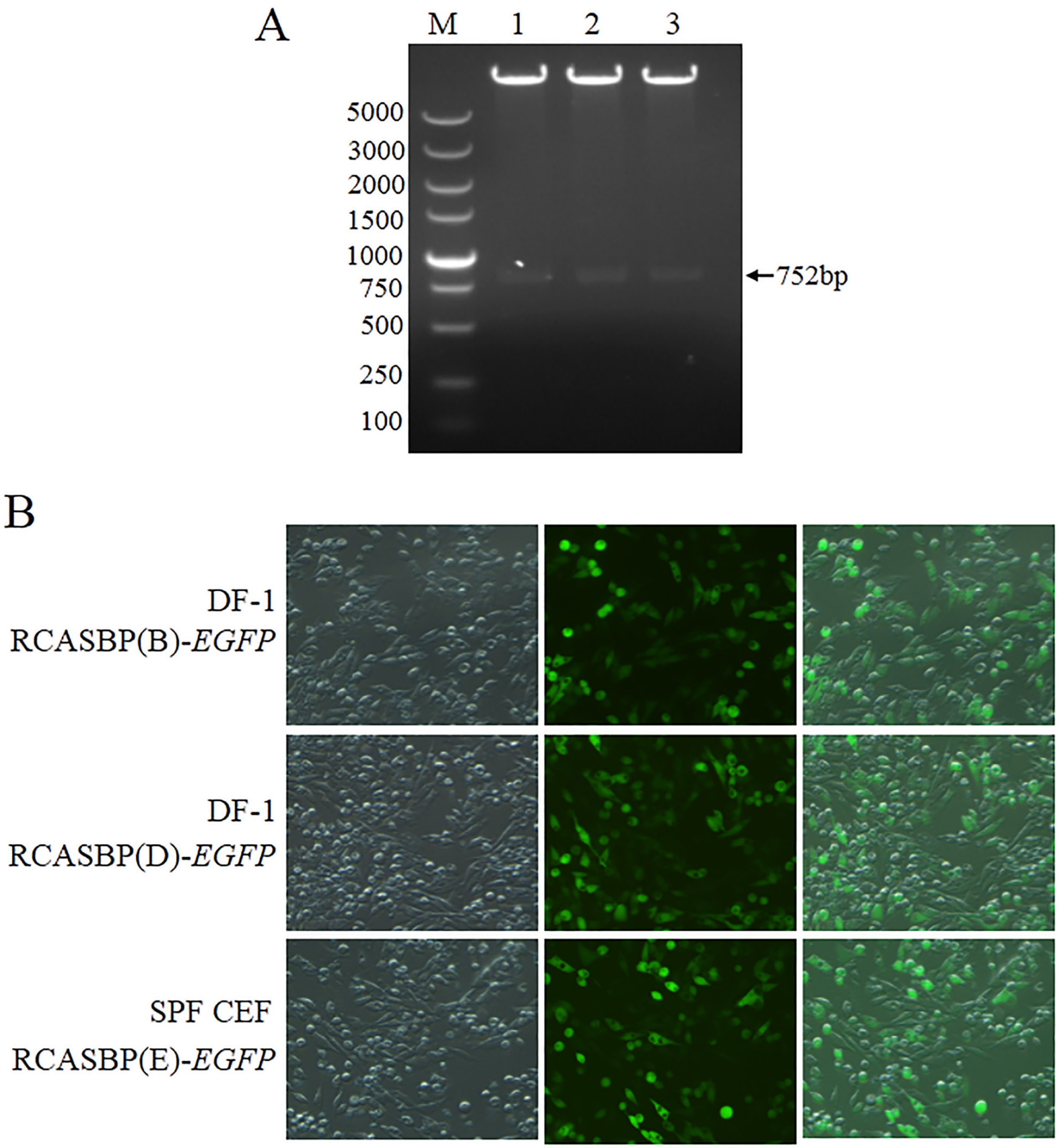

Supplementary Figure 2: Construction of subgroups B, D, and E ALV reporter vectors and viruses propagation. **(A)** Enzyme digesting identification using *Cla*I of the recombinant plasmids RCASBP(B)-EGFP (Lane 1), RCASBP(D)-EGFP (Lane 2), and RCASBP(E)-EGFP (Lane 3). M: DNA Marker 5000.The sizes of diagnostic GFP products are indicated on the left. **(B)** DF-1 cells were transfected with RCASBP(B)-EGFP and RCASBP(D)-EGFP plasmids DNA, and CEFs prepared from 10-day-old embryos of SPF chickens were transfected with RCASBP(E)-EGFP plasmids DNA. Representative images of producing GFP-transducing viruses were captured with a fluorescence microscope (Scale bars: 100 µm).

Supplementary Table 1 : Primers used for cloning and quantitative real-time PCR of *tvb* gene

Target	Primers	Sequence (5'-3')	Size(bp)	Accession No.
tvb-1	Forward	ACCAAACCCGTTCCTAACTC	1495	NC_006109.3
	Reverse	TGACCAAAGGAAGGAACCAT		
tvb-2	Forward	TCAGAACAGCCGTTTATTTGG	1486	
	Reverse	TGTCTGCCCTCTTCTTCACTG		
tvb-3	Forward	GAAAGCAGGCGTAATGGTGTCC	1625	
	Reverse	TGGGAGACAAACGCAGAGCAG		
tvb-4	Forward	CTCTGCGTTTGTCTCCCA	1055	
	Reverse	GCTCACCTCGTACTGGAAAT		
tvb-298	Forward	AGACGAGTACACCGAGTATCCA	214	AF161713.1
	Reverse	GCACGGAGCTAACCTCACTT		
tvb-exon-1-3	Forward	TCTTCGCGGAGGTTCAGT	170	AF161713.1
	Reverse	TGGATACTCGGTGTACTCGTCT		
tvb-exon10	Forward	TCCCAAAGTGGAAACCC	169	
	Reverse	CTGCTCTGCCAGATAAAGG		
chGAPDH	Forward	TGCCATCACAGCCACACAGAAG	123	NM_204305.1
	Reverse	TGCCATCACAGCCACACAGAAG		
tvb-mRNA	Forward	CC<u>CTCGAG</u>ATGCGCTCAGCTGCGCT	1107	AF161713.1
	Reverse	CC<u>AAGCTT</u>TCAGCTCACCTCGTACTG		

tvb-1, *tvb*-2, *tvb*-3 and *tvb*-4 = four primer pairs for cloning the whole genomic region of *tvb* gene; *tvb*-298 = primers for cloning a portion of *tvb* cDNA encompassing the c.298C>T mutation; *tvb*-exon-1-3 and *tvb*-exon10 = primers for cloning 5' and 3' end of the *tvb* cDNA, respectively, and were used for real-time PCR analysis, chGAPDH = primers for cloning partial cDNA of chicken glyceraldehyde-3-phosphate dehydrogenase (GAPDH), which was used as an internal control; *tvb*-mRNA = primers for cloning the entire coding sequence of *tvb* gene, the underlined sequences in the forward and reverse primers are the enzyme digest sites of *Xho*I and *Hind*Ш, respectively.

Supplementary Table 2 : Primers used for construction of SU(B)-rIgG, SU(D)-rIgG and SU(E)-rIgG

Target	Primers	Sequence (5'-3')	Size (bp)	Accession No.
SU(B)	Forward	CGAGCTCATGGATGTTCACTTACTCGAGCAGC	1044	HM446005.1
	Reverse	GGGGTACCTCGTTTATGTCTTACCCCTGTT		
SU(D)	Forward	CGAGCTCATGGATGTCCACTTACTCGAGCA	1041	D10652.1
	Reverse	GGGGTACCGCTTCGTTTGCGTCTTACA		
SU(E)	Forward	CGAGCTCATGGACGTCCATTTACTCGAGCA	1017	M12172.1
	Reverse	GGGGTACCTTTACGTCTTATACCTGTCCG		
rIgGFc	Forward	GGGGTACCCCCTCGACATGCAGCAAG	681	DQ402474.1
	Reverse	CGGGATCCTGATTTACCCGGAGAGCG		

SU(B), SU(D) and SU(E) = primers for cloning the SU coding region of subgroups B, D and E ALV env gene respectively, the underlined sequences in the forward and reverse primers are the enzyme digest sites of *Sac*I and *Kpn*I, respectively. rIgGFc = primers for cloning the IgG heavy chain of rabbit, the underlined sequences in the forward and reverse primers are the enzyme digest sites of *Kpn*I and *BamH*I, respectively.
